# Supplementary material for: 4-Mercaptobenzoic Acid Labeled Gold-Silver-Alloy-Embedded Silica Nanoparticles as an Internal Standard Containing Nanostructures for Sensitive Quantitative Thiram Detection
Source: Int J Mol Sci. 2019 Sep 29;20(19):4841. doi: 10.3390/ijms20194841 (PMC6801479; doi:10.3390/ijms20194841)
Supplement: Supplementary file 1 [file ijms-20-04841-s001.pdf]

International Journal of *Molecular Sciences*

4-Mercaptobenzoic Acid Labeled Gold-Silver-alloy-embedded Silica Nanoparticles as an Internal Standard Containing Nanostructures for Sensitive Quantitative Thiram Detection

Xuan-Hung Pham [1], Eunil Hahm [1], Kim-Hung Huynh [1], Byung Sung Son [1], Hyung-Mo Kim [1], Dae Hong Jeong [2] and Bong-Hyun Jun [1,*]

[1] Department of Bioscience and Biotechnology, Konkuk University, Seoul 143-701, Republic of Korea; phamricky@gmail.com (X.-H.P.); greenice@konkuk.ac.kr (E.H.); huynhkimhung82@gmail.com (K.-H.H.); imsonbs@konkuk.ac.kr (B.S.S.); hmkim0109@konkuk.ac.kr (H.-M.K.)

[2] Department of Chemistry Education, Seoul National University, Seoul 151-742, Republic of Korea; jeongdh@snu.ac.kr

* Correspondence: bjun@konkuk.ac.kr; Tel.: +82-2-450-0521

Supplementary materials:

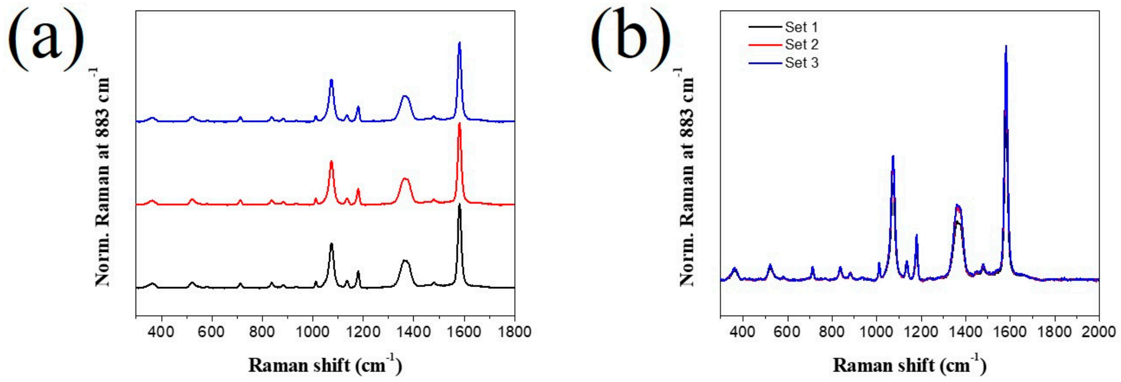

Figure S1. (**a**) Reproducibility and (**b**) repeatability of SERS signal of SiO2@Au@4-MBA@Ag NPs.

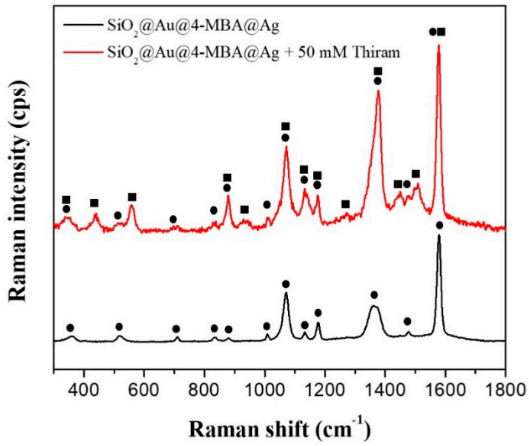

Figure S2. SERS spectra of SiO_2@Au@4-MBA@Ag nanoparticles in the presence and absence of 50-uM thiram.

International Journal of
Molecular Sciences

MDPI

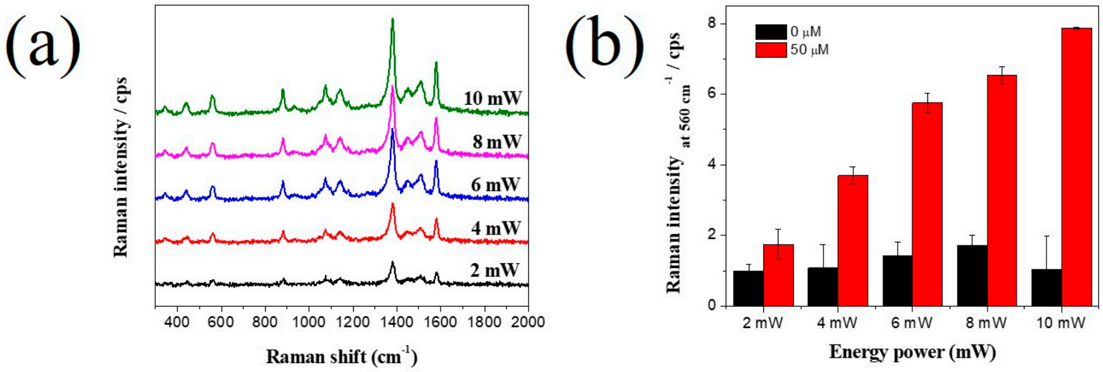

Figure S3. Effect of employed power energy on SERS signals of thiram detection: (**a**) SERS spectra and (**b**) SERS signal plot of SiO_2@Au@4-MBA@Ag nanoparticles in the presence of 50-uM thiram at employed power energy in the range of 2–10 mW.

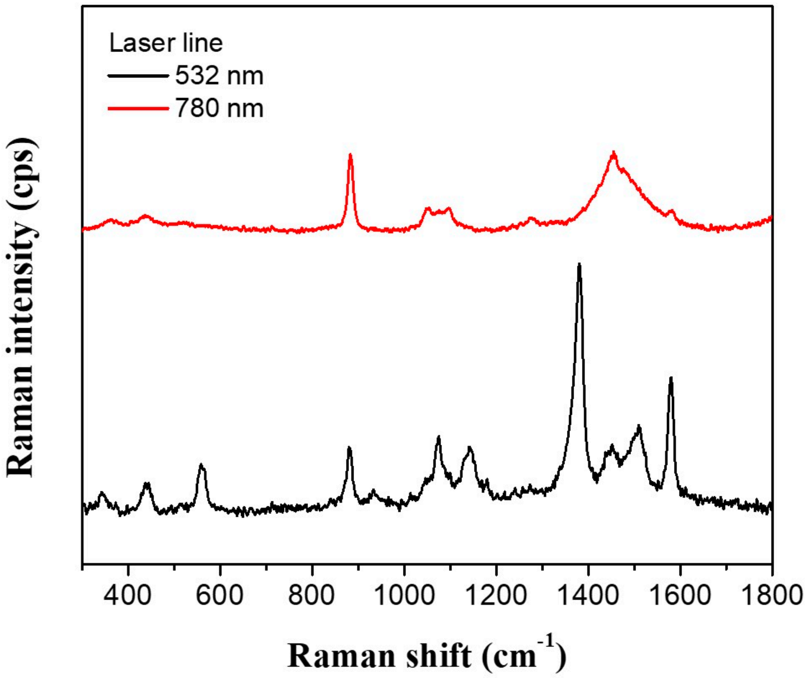

Figure S4. Effect of laser lines on SERS signals of thiram detection by SiO_2@Au@4-MBA@Ag nanoparticles in the presence of 50uM thiram at 10 mW.
